# Processes Underlying the Relation between Cognitive Ability and Curiosity with Academic Performance: A Mediation Analysis for Epistemic Behavior in a Five-Year Longitudinal Study

**DOI:** 10.3390/jintelligence10020023

**Published:** 2022-04-13

**Authors:** Patrick Mussel

**Affiliations:** Division for Personality Psychology and Psychological Assessment, Freie Universität Berlin, Habelschwerdter Allee 45, 14195 Berlin, Germany; patrick.mussel@fu-berlin.de

**Keywords:** cognitive ability, curiosity, academic performance, environmental enrichment, differential preservation hypothesis, mediation

## Abstract

Cognitive ability and curiosity are significant predictors of academic achievement; yet the processes underlying these relations are not well understood. I drew on ideas from the environmental enrichment hypothesis and the differential preservation hypothesis and hypothesized that epistemic behavior acts as a mediator. Longitudinal data were collected from 1964 individuals in three waves, spanning five years: cognitive ability and curiosity were assessed at time 1; epistemic behavior at time 2; at time 3, grade point average and highest degree of both secondary and tertiary academic education (if applicable) were obtained retrospectively via self-report. I found expected bivariate relations between all study variables, including a significant relation between cognitive ability and curiosity and significant relations of both of these variables with secondary academic performance. Epistemic behavior was related to curiosity and academic performance but, at odds with the hypothesis, did not mediate the relation between cognitive and personality variables and academic performance. It is concluded that the process underlying the behavioral consequences of cognitive ability and curiosity is not environmental enrichment.

## 1. Introduction

Cognitive ability and curiosity are two important antecedents of academic achievement (Anastasi 1982; Kashdan and Yuen 2007; Kuncel et al. 2004; Poropat 2009; Richardson et al. 2012; von Stumm et al. 2011). In terms of competences, they refer to latent characteristics (resources) underlying behavior responsible for knowledge acquisition and performance (Blömeke et al. 2015). However, the processes underlying these predictions are less well known. Here, I investigate the environmental enrichment hypothesis (Raine et al. 2002; Ziegler et al. 2012), according to which engagement in epistemic behaviors lead to an enrichment of the environment, thus providing more opportunity for the acquisition of knowledge.

### 1.1. Investment Theory

According to Cattell’s (1987) investment theory, crystallized intelligence develops out of fluid intelligence due to the investment of time in intellectual pursuit and knowledge acquisition. Up to adolescence, crystallized intelligence consists largely of the school curriculum as reflected in school grade point averages or scores on standardized tests such as the SAT, which Cattell (1987) referred to as historical crystallized intelligence. What crystallized intelligence constitutes after secondary education, however, differs largely between individuals or groups of individuals according to decisions made for certain educational, occupational, and leisure activities and the knowledge and expertise obtained in their chosen domains. For example, during tertiary education, the grade point average within a subject reflects crystallized intelligence (Ackerman 1996). Cattell’s (1987) general assumption of fluid intelligence predicting crystallized intelligence has received support. For example, measures of fluid intelligence have been found to predict secondary and tertiary academic performance (Anastasi 1982; Kuncel et al. 2004; Poropat 2009; Richardson et al. 2012) and trajectories in crystallized intelligence (Ghisletta and Lindenberger 2003; McArdle et al. 2000).

Cattell (1987) also considered interests as non-cognitive variables influencing the acquisition of knowledge by determining the orientation towards specific knowledge domains. However, the inclusion of personality traits was not considered until Ackerman (1996) proposed his theory of intelligence as process and as knowledge. Especially, he considered openness (Goldberg 1990) and typical intellectual engagement (Goff and Ackerman 1992) as variables which reflect individual differences in the tendency to engage in and enjoy effortful cognitive processing. These variables, together with others such as curiosity (Litman 2005), have been referred to as investment traits (von Stumm and Ackerman 2013). The term emphasizes that these personality traits influence when, where, and how people invest time and effort into knowledge acquisition. According to the theoretical intellect model (Mussel 2013a), these refer to individual differences in the propensity to think, learn, and create and influence seeking out and conquering intellectually challenging situations. Investment traits have been found to be related to fluid intelligence (Ackerman and Heggestad 1997; Fleischhauer et al. 2010; von Stumm 2013; von Stumm and Ackerman 2013) and academic performance (Kashdan and Yuen 2007; Richardson et al. 2012; von Stumm et al. 2011).

### 1.2. Environmental Enrichment

The process underlying these relations might be attributed to cognitive environmental enrichment. This term was chosen by Raine et al. (2002), who found that stimulation-seeking 3-year-olds showed higher levels of fluid intelligence and scholastic and reading ability at age 11. The authors theorized that so called “stimulation seekers” search for opportunities for engaging with cognitively challenging stimuli, thereby enriching their environment, providing a playground for utilizing their cognitive abilities, and obtaining new knowledge. In line with these results, parental stimulation was found to be associated with cognitive development (Tucker-Drob and Harden 2012). Similarly, Muentener et al. (2018) found exploratory behavior of children to be associated with cognitive development, and Silvia et al. (2016) found effects of music training on cognitive abilities.

The openness, fluid, and crystalized intelligence (OFCI) model by Ziegler et al. (2012, 2015) applied the idea of environmental enrichment to investment traits. The model proposes that openness predicts fluid and crystallized intelligence (the latter mediated via fluid intelligence) as individuals high compared to low on openness to experience seek more learning opportunities, thereby enriching their environment according to their personality.

While intuitively appealing, direct empirical evidence using behavioral indicators for the assumed underlying processes is sparse. Kraaykamp and van Eijck (2005) report relations between openness and reading as a leisure activity. Trapp et al. (2019) report cross-sectional evidence for a mediation effect of reading and calculating as behavioral indicators of environmental enrichment on the relation between openness and fluid intelligence. Trapp and Ziegler (2019) replicated this cross-sectional effect and additionally found a prospective effect of openness on reading three years later.

Additional evidence for the environmental enrichment hypothesis comes from the aging literature. Here, trajectories in cognitive functioning are assumed to depend on engagement in physical, social, and intellectual activities, labeled as the differential preservation hypothesis (Salthouse 1991). According to the “use-it-or-lose-it” principle, less decline in cognitive abilities is assumed to be a function of more frequent participation in cognitively demanding activities (Salthouse 2006). Empirical evidence for this hypothesis is mixed. While engagement has repeatedly been found to be associated with both investment traits and cognitive functioning (e.g., Romeiser et al. 2021; Salthouse and Mitchell 1990), evidence for a moderating or mediating influence of engagement on trajectories in cognitive functioning is often lacking. For example, von Stumm (2012) found that the investment trait need for cognition was associated with cognitive engagement. However, engagement did not mediate age-related effects on levels of fluid and crystallized intelligence, thus disconfirming the environmental enrichment hypothesis.

### 1.3. The Present Study

In the present longitudinal study, I investigate the environmental enrichment hypothesis with regards to the relation between fluid intelligence and curiosity on one hand and secondary and tertiary academic performance as indicator of knowledge attainment on the other. I predict that epistemic behavior partially mediates (1) the relation between cognitive ability and academic performance and (2) the relation between curiosity and academic performance. Thereby, epistemic behavior is considered as cognitively challenging behavior during work or leisure activities, such as reading, thinking, or learning (see for example Trapp and Ziegler 2019).

The first hypothesis is based on Cattell’s (1987) assumption that crystallized intelligence develops out of fluid intelligence due to the investment of time in intellectual pursuit and knowledge acquisition. Individuals with higher compared to lower cognitive abilities might seek cognitively challenging situations more often as they expect to succeed in such situations, as proposed by the environmental success hypothesis (Ziegler et al. 2012); additionally, seeking such situations allows the investment of fluid abilities in epistemic behavior and the acquisition of new knowledge. Thus, among other processes such as quicker understanding and better memory, the relation between fluid intelligence and an indicator of crystallized intelligence such as academic grades would be mediated by the frequency of engaging in epistemic behaviors.

The second hypothesis proposes that individual differences in the tendency to environmental enrichment are reflected in investment traits such as curiosity. In particular, it assumes that the process underlying the relation between curiosity and an indicator of crystallized intelligence can be explained by an enriched environment: Individuals high compared to low on curiosity more often engage in epistemic behavior, which provides opportunity to acquire new knowledge. Thus, the relation between curiosity and academic grades would be mediated by the frequency of engaging in epistemic behaviors.

## 2. Materials and Methods

### 2.1. Panel and Procedure

Data were collected from participants of the German Personality Panel (GePP, Mussel 2021). The panel was established by contacting individuals who formerly (in 2016 or 2017) participated in an online career counseling test (berufsprofiling.de). The counseling is free of charge, and participants agreed to be contacted later. According to the scope of the counseling, most individuals presumably had prepared their first career decision by that time. From 11,816 individuals who were contacted later via email, a total of 1964 people participated in the study and agreed that their data, obtained during the counseling, may be used. I refer to this measurement occasion as time 1 (T1).

Data on the second measurement occasion time (T2) were collected in September 2018. I contacted former participants, as noted above, and invited them to participate in a research panel study. Individuals who agreed took an online test that lasted about 35 min. In return, they received feedback on their test results and an Amazon voucher worth €5.00. A total of 1679 individuals participated at T2.

In November 2021, all active panel members at that time (*N* = 1601) were contacted again. Active panel members were individuals who formerly provided full data on at least one measurement occasion (including but not limited to T2) and had not in the meantime decided to opt out. A total of 465 individuals agreed to participate. All of them had valid data on T1, and 377 also on T2. They took a test battery, similar to T2, taking approximately 35 min, and received a €10 voucher from “wunschgutschein.de” and feedback on their results. Data from this measurement occasion are referred to as time 3 (T3).

### 2.2. Sample

The total sample size is *N* = 1965 (with data on at least one measurement occasion). At the time of T1, most panel members were between 14 and 22 years old (mean age: *M* = 17.3; *SD* = 2.3), 65% identified themselves as female and 35% as male. According to a subsample from whom year of graduating from school was assessed (*N* = 419, obtained at T3), 74% were still at school when taking the career counseling. The test was applied in German language.

### 2.3. Measures

A full description of all measures obtained at all measurement occasions is available online (Mussel 2021). Data from the following measures are reported in the present study:

Curiosity was assessed using the 10-item Work-Related Curiosity Scale (WORCS; Mussel et al. 2012). An example item is “I am eager to learn”. Responses are given on a 7-point Likert scale ranging from “does not apply at all” (1) to “partly” (4) to “fully applies” (7). The test was applied at T1, T2, and T3. In the present study, the reliability coefficient at T1 was ω = 0.90 and retest reliabilities were *r*_xx_ = 0.57 and 0.53 with T2 and T3, respectively.

Cognitive ability was assessed with three short indicators assessing numeric, figural, and verbal abilities (HR Diagnostics n.d.). Tests were assessed at T1, T2, and T3. For numerical abilities, a 10-item number series test was used. Each item contains of a series of numbers that must be completed. Answers are given as free text, and the correct answer is scored as “1”. In the present study, the reliability coefficient at T1 was ω = 0.80 and the retest reliabilities were *r*_xx_ = 0.66 and 0.70 with T2 and T3, respectively.

For figural abilities, a 20-item matrix test was used. Each item consists of a classical 3 × 3 matrix, with the last element substituted by a question mark. Participants must choose an object from a list of six objects that logically fits such that the underlying role is preserved. Correct answers are scored as “1”. In the present study, the reliability coefficient at T1 was ω = 0.75 and retest reliability was *r*_xx_ = 0.59 with both T2 and T3. For verbal abilities, a 28-item measure to assess vocabulary was used. Each item consists of five words, four of which do not exist. Here, participants must choose the word that exists. Correct answers are scored as “1”. In the present study, the reliability coefficient at T1 was ω = 0.86 and the retest reliabilities were *r*_xx_ = 0.75 and 0.65 with T2 and T3, respectively.

Indicators for all three subtests were standardized and subsequently aggregated. For the aggregated score, the reliability coefficient across the three subtests was ω = 0.90 and the retest reliabilities were *r*_xx_ = 0.77 and 0.75 with T2 and T3, respectively.

For the assessment of environmental enrichment, I used four indicators of epistemic behavior at work and during leisure time. All four indicators were applied at T2 and T3.

First, epistemic behavior at work was assessed using 20 items from the PIAAC international codebook (Rammstedt et al. 2013). These items assess skill use on the job regarding literacy and numeracy. An example item is “reading instructions and manuals”. Responses are given on a 5-point Likert scale with the response options “never” (1), “less than once a month” (2), “at least once a month, but not once a weak” (3), “at least once a week, but not daily” (4), and “daily” (5). In the present study, the reliability coefficient at T2 was ω = 0.90 and the retest reliability from T2 to T3 was *r*_xx_ = 0.31 (note that low correlations may also reflect changes in work and leisure behavior across the three-year period, for example when graduating from high school or university).

Second, work characteristics with regards to epistemic behavior were assessed using the 27-item instrument assessing curiosity activating work characteristics (Mussel and Spengler 2015). The instrument assesses job demands, job constraints, and job distractors, each on the task, social, and organizational level, according to trait activation theory (see Tett and Burnett 2003). An example item is “My current education/occupation requires creative solutions for new problems”. Responses are given on a 7-point Likert scale ranging from “does not apply at all” (1) to “partly” (4) to “fully applies” (7). In the present study, the reliability coefficient at T2 was ω = 0.80 and the retest reliability from T2 to T3 was *r*_xx_ = 0.31.

Third, epistemic leisure activities were assessed with five ad-hoc developed items (e.g., reading books, attending advanced training; acquiring new knowledge). Responses were given on a 5-point Likert scale with the response options “never” (1), “seldom” (2), “sometimes” (3), “often” (4), and “very often” (5). In the present study, the reliability coefficient at T2 was ω = 0.72 and retest reliability from T2 to T3 was r_xx_ = 0.48.

Fourth, non-epistemic leisure activities were assessed with three ad-hoc developed items (e.g., watching TV/streaming; sport). Responses are given on a 5-point Likert scale with the response options “never” (1), “seldom” (2), “sometimes” (3), “often” (4), and “very often” (5). In the present study, the reliability coefficient at T2 was ω = 0.23 and the retest reliability from T2 to T3 was r_xx_ = 0.45 (note that these leisure activities are not supposed to assess a single underlying dimension but are all theoretically associated with non-epistemic behavior).

The four indicators of epistemic behavior underlying environmental enrichment are not expected to reflect a unidimensional latent variable. However, they are all theoretically chosen or developed to assess the intended construct. In the present study, the reliability coefficient across the four indicators at T2 was ω = 0.25 and the retest reliability from T2 to T3 was *r*_xx_ = 0.43.

Grades were assessed via self-report at T3. I asked participants to report their highest secondary school degree using a drop-down field with four options: “no school degree (thus far)”; “Hauptschule”; “Realschule”; “Hochschulreife (Abitur)” (according to the German school system). If applicable, I asked for year of graduation from high school and their grade point average as an indicator of secondary school performance. As an indicator of tertiary school performance, I asked them to report their highest occupational or university degree using a drop-down field with the four options “no occupational/university degree”; “vocational training”; “Bachelor”; and “Master”. If applicable, I asked the year in which they received their degree and the final grade point average. Grades were assessed at T3.

Type of academic degree varies strongly in complexity, thus making academic grades from different school types and different educational/university institutions hardly comparable. I used the method provided by Görlich and Schuler (2007) to correct for level differences between types of school/academic degree. For school degree, the authors provide estimates reflecting level differences of −1.8 for “Hauptschule”; −1.1 for “Realschule”; and 0.8 for “Hochschulreife/Abitur”. These values are added to the transformed grades (thus higher values reflecting better performance). For occupational/university degree, no recommendations were available. Therefore, level differences had to be estimated according to mean differences in z-standardized cognitive ability at T3 as −0.22 for “vocational training” and 0.17 for “Bachelor/Master” (the latter two had to be aggregated due to the small number of individuals with a master’s degree). These estimated level differences between degrees were analogously added to the transformed grade point average, resulting in the grade being corrected for type of academic degree.

Grades for secondary school were reported by *N* = 421 individuals; *N* = 143 reported to have received a tertiary degree. The correlation between grades (corrected for different types of degree) from secondary and tertiary education was *r* = 0.40.

For the assessment of careless responding, a single self-reported item asking participants whether they had worked on the test carefully (with two options: yes and no) was obtained. Participants were instructed that their answer would not affect obtaining the financial incentive. The careless response indicator was obtained at T2 and T3.

### 2.4. Careless Responding, Missingness, and Attrition

At T2, *N* = 41 (2.4%) and at T3, *N* = 4 (0.9%) answered with “no” and were subsequently excluded from further analysis. Additionally, scores from measures with more than 20% of missingness were excluded from the analysis. Accordingly, *N* = 28 values were missing for cognitive ability, *N* = 306 for curiosity and *N* = 444 for epistemic behavior. The final sample size for all measures and measurement occasions can be seen in Table 1.

As noted above, the total sample consists of *N* = 1965 individuals, 1719 of whom participated at least at two measurement occasions. The number of cases with full data on all variables pertaining to a particular analysis were *N* = 279 (for curiosity, epistemic behavior, and secondary grades); *N* = 312 (for cognitive ability, epistemic behavior, and secondary grades); *N* = 113 (for curiosity, epistemic behavior, and tertiary grades); and *N* = 117 (for cognitive ability, epistemic behavior, and tertiary grades).

Probabilities of non-missingness at T2 and T3 were independent of levels of curiosity at T1 (r = 0.02 and 0.01), and probability of non-missingness at T3 was unrelated to levels of curiosity at T2 (r = −0.01). For cognitive ability, there was a small effect for higher probability for participation according to higher compared to lower levels in cognitive abilities on previous measurement occasions (r = 0.19/0.14/0.14). Finally, probabilities of non-missingness at T3 were unrelated to levels of epistemic behavior at T2.

### 2.5. Statistical Power

Due to the longitudinal design, obtaining a desired sample size according to an à priori power analysis was not possible. Post hoc power for the obtained sample size was calculated with the R package bnem according to Zhang (2014) for a medium effect of *a* = *b* = 0.39 according to the recommendations of Zhang (2014) as well as *a* = *b* = 0.20 according to the recommendations of Gignac and Szodorai (2016) for effect size in individual differences. Note that the latter is closer to effect size estimates for predicting academic performance (Kuncel et al. 2004; Poropat 2009; Richardson et al. 2012; von Stumm et al. 2011). Effect sizes were calculated for the sample with full data on all relevant variables, leading to a conservative estimate of statistical power (power can be enhanced by including cases with missing data, but power estimated for specific patterns of missingness are, to my knowledge, not available).

For predicting tertiary academic performance, the statistical power was 0.97/0.96 for cognitive ability/curiosity for *a* = *b* = 0.39 but only 0.52/0.49 for *a* = *b* = 0.20. Thus, only larger effects can be detected with acceptable power. For predicting secondary academic performance, the statistical power was 1.00 for both cognitive ability and curiosity for *a* = *b* = 0.39 and 0.97/0.96 for *a* = *b* = 0.20. Thus, results for secondary performance can also detect medium effect size with higher power; however, causal inferences must be interpreted with caution as some participants obtained their secondary degree prior to T2.

### 2.6. Statistical Analyses

All data analyses were conducted in R-Studio 2021.09.1 on R 4.1.2 using the packages *psych* (Revelle 2020) and *lavaan* (Rosseel 2012). Prior to hypothesis testing, I tested item overlap between the curiosity measure and the indicators of epistemic behavior (Lemery et al. 2002; Lengua et al. 1998). Therefore, I ran four separate exploratory principal axis factor analyses. For each analysis, the 10 items of the Work-Related Curiosity Scale were analyzed, together with the items of one of the four indicators of epistemic behavior, respectively.

**Figure 1:**
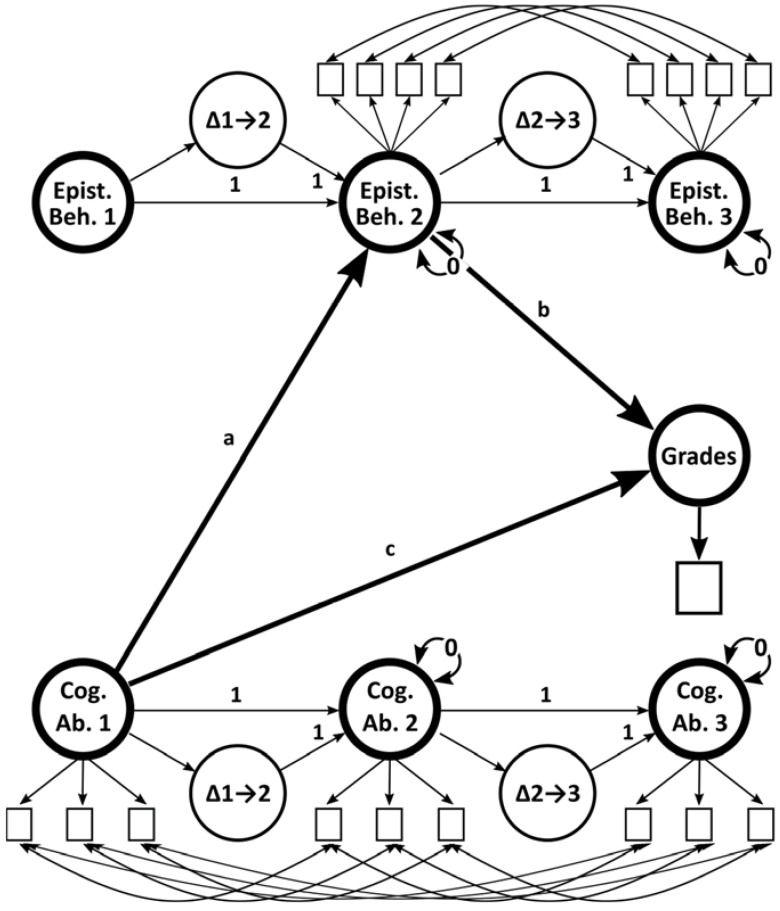
Schematic figure of the latent growth model with mediation effects. Epist. Beh.: epistemic behavior; Cog. Ab: cognitive ability. Epistemic behavior was not measured at time 1 and was included as a phantom variable. In a separate model, curiosity (instead of cognitive ability) was used as predictor.

For the main analysis, given the longitudinal structure of the data, a hierarchical latent change score model (Horn and McArdle 1992; Mund and Nestler 2019; Newsom 2017) with mediation effects and robust maximum likelihood estimator was specified according to Figure 1 and analyzed with the *sem* function of the *lavaan* package. Data on the full sample (N = 1965) were analyzed, and missingness was handled via full information likelihood (Enders and Bandalos 2001).

The model was built stepwise. First, measurement invariance was tested for cognitive ability across the three measurement occasions. For the configural model, the three cognitive tests served as manifest indicators on each measurement occasion. Latent variances were fixed to 1 and latent variables were allowed to correlate. I subsequently tested fit for weak measurement invariance by fixing the regression parameters across measurement occasions. Next, fit for assumed strong measurement invariance was tested by additionally fixing the means of the manifest variables across measurement occasions.

Second, measurement invariance was tested for epistemic behavior. The four indicators of epistemic behavior served as manifest variables on measurement occasions T2 and T3. As no indicator was available at T1, a phantom variable was included with a mean of zero and a variance of one (Harring et al. 2017; Rindskopf 1984). Subsequently, fit for assumed weak and strong measurement invariance was tested.

Third, based on the results of measurement invariance testing, cognitive ability and epistemic behavior were jointly added to the model. Forth, in separate models for secondary and tertiary academic performance, grades were included in the model as a latent single indicator variable. The error variance of the self-reported grades was assumed to be 0.10 (equaling a reliability of 0.90), and the regression parameter was fixed to one. The latent variable representing grades was regressed on latent cognitive ability at T1, establishing the direct effect for predicting grades according to levels of cognitive ability. Finally, the indirect effect via epistemic behavior was added by additionally regressing latent grades on latent epistemic behavior at T2 and regressing latent epistemic behavior at T2 on latent cognitive ability at T1.

For curiosity as predictor, the ten items of the Work-Related Curiosity Scale were aggregated to three parcels by optimizing equal loadings on the latent factor and equal means across parcels. The following steps were performed analogously to the model for cognitive ability.

### 2.7. Preregistration

The present study was preregistered on 29 October 2021, prior to the collection of data on T3, on OSF (https://osf.io/th86q/; accessed on 12 April 2022). Deviations from the preregistration are detailed in Appendix A.

## 3. Results

Regarding precursory analyses testing item overlap between the curiosity scale and the measures for epistemic behavior, I found that in all of the four exploratory factor analyses the items of the Work-Related Curiosity Scale loaded on their respective factor. Additionally, for PIAAC, epistemic leisure activities, and non-epistemic leisure activities, the items of these indicators also loaded on their respective factor. For work characteristics with regards to epistemic behavior, items pertaining to job constraints and job distractors loaded on their respective factor; yet all items pertaining to job demands loaded together with the items of the Work-Related Curiosity Scale. As this pattern might be due to the relative independence of job demands, distractors, and constraints, I re-ran the analysis excluding distractors and constraints. Indeed, for this analysis, all items pertaining to job demands loaded on their respective factor. Thus, item overlap between predictor and mediator can be ruled out.

Bivariate correlations are depicted in Table 1. In line with the literature, cognitive ability and curiosity were positively correlated (*r* = 0.27 for T1). Epistemic behavior, obtained at T2, was significantly correlated with curiosity, but not with cognitive ability.

Regarding academic performance, a differential pattern emerged for secondary and tertiary education. Secondary academic performance obtained at T3 was significantly predicted by both cognitive ability and curiosity, with a larger correlation coefficient for the former compared to the latter (*r* = 0.39 vs. 0.20 for T1). In addition, epistemic behavior at T2 significantly predicted secondary academic performance (*r* = 0.15). In contrast, none of the three constructs was significantly associated with tertiary academic performance.

For cognitive ability, the configural model showed a good fit (χ^2^ = 45, *df* = 15, CFI = 0.993, RMSEA = 0.03, GFI = 0.93, AGFI = 0.74, SRMR = 0.02). The model reflecting weak measurement invariance revealed a similar fit (χ^2^ = 96, *df* = 21, CFI = 0.982, RMSEA = 0.05, GFI = 0.93, AGFI = 0.83, SRMR = 0.05), even though the change in CFI marginally exceeded the recommended boundary (ΔCFI ≤ 0.01; Cheung and Rensvold 2002). When subsequently testing for strong measurement equivalence, change in CFI and modification indices indicated that means for two of the nine indicators should be freed, resulting in a model with adequate fit (χ^2^ = 131, *df* = 23, CFI = 0.975, RMSEA = 0.05, GFI = 0.94, AGFI = 0.85, SRMR = 0.06). Therefore, partial strong measurement invariance across measurement occasions could be assumed.

Regarding epistemic behavior, the configural model showed good fit (χ^2^ = 19, *df* = 15, CFI = 0.994, RMSEA = 0.01, GFI = 0.99, AGFI = 0.98, SRMR = 0.03). Model fit changed (ΔCFI > 0.01) when assuming weak and strong measurement equivalence. According to modification indices, one of the four regression parameters and two of the eight means were freed. The model assuming partial strong measurement equivalence showed good fit (χ^2^ = 24, *df* = 18, CFI = 0.991, RMSEA = 0.02, GFI = 0.99, AGFI = 0.98, SRMR = 0.03). Thus, partial strong measurement equivalence could be assumed.

Next, secondary academic grades were included in the measurement model for cognitive ability regressed on latent cognitive ability at T1 (χ^2^ = 162, *df* = 28, CFI = 0.970, RMSEA = 0.05, GFI = 0.93, AGFI = 0.84, SRMR = 0.06). The regression parameter was significant (standardized β = 0.51, *p* < 0.001), indicating a significant prediction of secondary academic performance according to levels of cognitive ability. Finally, the measurement model for epistemic behavior was added to the model, including the path from latent cognitive ability at T1 to latent epistemic behavior at T2 and the path from latent epistemic behavior at T2 to grades (χ^2^ = 377, *df* = 115, CFI = 0.952, RMSEA = 0.04, GFI = 0.93, AGFI = 0.89, SRMR = 0.08). Parameter estimates for the final model can be seen in Table 2. While the direct effect of cognitive ability at T1 on grades remained significant, neither the regression from cognitive ability on epistemic behavior nor the regression from epistemic behavior on grades and, subsequently, the indirect effect approached significance. Thus, the mediation effect was not supported.

The model predicting tertiary (rather than secondary) academic performance yielded a similar fit (χ^2^ = 345, *df* = 115, CFI = 0.957, RMSEA = 0.03, GFI = 0.93, AGFI = 0.89, SRMR = 0.08). However, in line with bivariate correlations, cognitive ability was not a significant predictor of tertiary performance. Additionally, when including the indirect effect via epistemic behavior, there was no evidence for a mediation effect (see Table 2 for regression estimates).

In a separate set of analyses, I analogously tested curiosity (rather than cognitive ability) as a predictor of academic performance. Regarding measurement invariance, the configural model for curiosity revealed excellent fit (χ^2^ = 10, *df* = 15, CFI = 1.0, RMSEA = 0.00, GFI = 1.00, AGFI = 0.99, SRMR = 0.01). The model reflecting weak measurement invariance revealed a similar fit (χ^2^ = 70, *df* = 21, CFI = 0.991, RMSEA = 0.04, GFI = 0.99, AGFI = 0.97, SRMR = 0.08); the differences were within ΔCFI ≤ 0.01. For establishing strong measurement invariance, it was necessary to free one of the nine means of the manifest variables. Thus, partial strong measurement invariance was achieved with satisfactorily model fit (χ^2^ = 92, *df* = 24, CFI = 0.988, RMSEA = 0.04, GFI = 0.99, AGFI = 0.97, SRMR = 0.08).

Next, grades were added to the measurement model for curiosity and regressed on latent curiosity at T1 (χ^2^ = 70, *df* = 29, CFI = 0.993, RMSEA = 0.03, GFI = 0.99, AGFI = 0.98, SRMR = 0.08). The regression parameter from latent grades on latent curiosity at T1 was significant (β = 0.25; *p* < 0.001). When additionally including the measurement model for epistemic behavior, including the indirect effect of curiosity at T1 on grades via epistemic behavior, the model fit dropped (χ^2^ = 360, *df* = 116, CFI = 0.963, RMSEA = 0.03, GFI = 0.97, AGFI = 0.94, SRMR = 0.07). The total effect on grades remained significant (see Table 2). The effect of curiosity at T1 on epistemic behavior at T2 was significant, but not the effect for epistemic behavior at T2 on grades. Correspondingly, the indirect effect was not significant, thus yielding no support for the mediation hypothesis.

When predicting tertiary (rather than secondary) academic performance (χ^2^ = 72, *df* = 29, CFI = 0.992, RMSEA = 0.03, GFI = 0.99, AGFI = 0.98, SRMR = 0.08), the effect of curiosity at T1 on grades was not significant. When additionally including the measurement model for epistemic behavior (χ^2^ = 346, *df* = 116, CFI = 0.965, RMSEA = 0.03, GFI = 0.97, AGFI = 0.94, SRMR = 0.07), the effect of curiosity at T1 on epistemic behavior at T2 remained significant (see Table 2). However, neither curiosity nor epistemic behavior predicted tertiary grades.

### Exploratory Analyses

The mediation analyses were repeated separately for each of the four indicators for epistemic performance. Therefore, the models were adjusted by including latent epistemic behavior at time 2 and 3 being indicated by only one single manifest epistemic behavior indicator. For a Bonferroni adjusted *p*-level of *p* = 0.0125, it turned out that the indirect effect of predicting tertiary academic performance according to levels in cognitive ability was mediated by leisure epistemic behavior (β = 0.02; *p* < 0.001).

The mediation analysis was also repeated by excluding participants who indicated that they received their academic degree in or before 2018. Accordingly, *N* = 297 (71%) of the cases were excluded for secondary academic performance and *N* = 18 (13%) of the cases were excluded for tertiary performance. Two results changed: First, the direct effect of curiosity on secondary academic performance was no longer significant (β = 0.17; *p* = 0.19). Second, for the model predicting tertiary performance via cognitive ability, the formerly non-significant direct effect was now significant (β = 0.26; *p* = 0.02). Regarding the hypotheses-relevant indirect effect, no changes occurred.

As suggested by an anonymous reviewer, educational attainment, which was used to correct grades for differences in complexity according to academic type, could be analyzed separately. Thus, analyses were repeated for standardized secondary (school degree, coded as −1.8 for “Hauptschule”; −1.1 for “Realschule”; and 0.8 for “Hochschulreife/Abitur”) and tertiary (i.e., educational degree, coded as −0.22 for “vocational training” and 0.17 for “Bachelor/Master”) educational attainment. Cognitive ability significantly predicted both secondary (β = 0.47; *p* < 0.001) and tertiary (β = 0.24; *p* = 0.01) educational attainment, but there was no mediation effect of epistemic behavior (β = 0.00/0.02; *p* = 0.84/0.93). Curiosity was unrelated to secondary educational attainment (β = 0.11; *p* = 0.09), but significantly predicted tertiary educational attainment (β = 0.29; *p* = 0.004). The indirect effect was not significant for either of the two criteria.

## 4. Discussion

The present study aimed to investigate the processes underlying the relation between dispositions and important life outcomes with the goal of enhancing our understanding of personality traits and setting the ground for potential interventions. Cognitive ability and curiosity are two established predictors of academic performance. However, the processes underlying these relations are largely unknown. In the present study, following the ideas of the environment enrichment and the differential preservation hypothesis, I set out to test the hypothesis whether individual differences in epistemic behavior function as a mediator.

Environment enrichment is a broad concept that has been investigated in human and non-human animals. It describes physical, psychological, and social features of a situation and encompasses non-epistemic (e.g., activity) and epistemic (e.g., learning) behaviors (van Praag et al. 2000). In line with an influence of environmental features on the organism is the concept of neural plasticity, proposing that the capacity of the nervous system modifies itself according to experience (Von Bernhardi et al. 2017), and the concept of epigenetics, according to which behaviors and environment can cause stable alterations in gene expression (Jaenisch and Bird 2003). Accordingly, trajectories in cognitive functions including knowledge acquisition might be influenced by corresponding behavior and experiences.

According to this reasoning, I proposed that individuals with higher compared to lower levels in cognitive ability and curiosity seek out cognitively stimulating and challenging situations which create a space for applying their fluid ability and, ultimately, acquiring new knowledge. At odds with these hypotheses, results from mediation analysis provided no support for the environmental enrichment hypothesis. First, the relation between fluid intelligence and academic performance was not mediated by epistemic behavior. This hypothesis was deduced from Cattell’s (1987) investment theory assuming that the influence of fluid intelligence on crystallized intelligence is a function of time invested in intellectual pursuit. However, while fluid intelligence and curiosity were, in line with the literature, significantly related (Ackerman and Heggestad 1997), epistemic behavior as a behavioral indicator of environmental enrichment was, at odds with the literature, unrelated to fluid intelligence (Vinkhuyzen et al. 2012).

Second, regarding curiosity, I replicated prior studies which consistently found relations between indicators of cognitive engagement, epistemic behavior, and cognitive performance, but no support for the mediation hypothesis (Soubelet 2011; von Stumm 2012). Curiosity is the desire to know, to see, or to experience that motivates exploratory behavior, information seeking, and learning (Berlyne 1954; Litman 2005; Loewenstein 1994; Mussel 2013b). In line with this definition, I found that individuals with high compared to low levels in curiosity indeed more often seek cognitively challenging situations (Trapp and Ziegler 2019). However, such behavior nonetheless does not seem to be the mechanism which underlies relations of curiosity with cognitive performance indicators.

Thus, it seems worthwhile to consider alternative hypotheses to environmental enrichment to understand the processes behind the relations between cognitive and personality variables and knowledge attainment. According to the ABCDs of personality (Revelle 2008), personality traits describe individual differences in affect, behavior, cognition, and desire/motivation. Investigating the processes underlying the relations between personality traits and important life outcomes requires the disentanglement of the elements characterizing a trait that are responsible for outcomes. As an alternative to behavioral “B” differences it has been proposed that the influence of investment traits on knowledge attainment might be rather due to constructing experiences in a cognitively stimulating manner (von Stumm 2012; von Stumm 2013), thereby shifting the focus to the level of cognitions “C”. For example, DeYoung et al. (2007) proposed that the domain of openness contains aspects of self-estimated intelligence (being clever; being smart); thus, relations of openness with cognitive criteria might be driven by differences in abilities which are reflected in personality measures. Alternatively, meta-cognitions and self-regulatory strategies have been proposed to underly the relation between investment traits and performance (Grass et al. 2019). Finally, it has been proposed that the relation between investment traits and performance might be driven by individual differences in basic cognitive functions (Gärtner et al. 2021). Regarding the latter, Mussel et al. (2016) showed that individuals who are high compared to low on the need for cognition invest higher levels of cognitive effort, assessed via theta power in the EEG, in complex compared to simple tasks, which subsequently predicted performance in a cognitive task.

In the present study and in conflict with the existing literature (Poropat 2009; Richardson et al. 2012), tertiary academic performance was not significantly associated with either cognitive ability or curiosity, which hampers investigation of environmental enrichment as the underlying process of this criterion. A potential reason for the lack of relation might be the diverse sample of the present study. Compared to studies which examine a cohort of students from a particular university and subject, participants of the GePP were recruited in the course of career counseling. Thus, reported grades might stem from all kinds of tertiary education. While a broad adjustment for vocational training versus university studies was applied, there is still plenty of variance within these two categories, ranging from warehouse clerk to mechatronic; from primary school pedagogy to physics and law studies; and from universities of applied science to state universities. However, exploratory analyses suggested that cognitive ability is related to tertiary academic performance in a subsample of individuals with a full prospective design and that both cognitive ability and curiosity predicted the criterion of tertiary educational attainment. Still, for neither of these additional analyses could a mediation effect for epistemic behavior be found.

Acknowledging these challenges and the rather small sample size of participants who had (yet) finished a tertiary education, secondary GPA was chosen as a second criterion. Regarding the latter, a larger number of participants reported their secondary grades. Additionally, curricula are more standardized in secondary compared to tertiary education. As a drawback, the design for secondary academic performance was not fully longitudinal as a large part of the sample reported that they had received their degree prior to T2. Thus, epistemic behavior as assessed at T2 only reflects environmental enrichment in relation to secondary academic performance to the degree that it indicates, as a variable with some degree of temporal stability, epistemic behaviors while still at school (for example when pursuing similar leisure activities before and after graduating).

The mediator of epistemic behavior was broadly conceptualized by including work and leisure activities and operationalized via four indicators. Thus, results might be influenced by the relevance of these behavioral indicators for the domain of academic performance. For example, frequent engagement in epistemic leisure activities such as going to the museum might even interfere with epistemic behavior related to academic performance (such as studying). However, at odds with this reasoning, post-hoc comparisons showed that only for epistemic leisure activities was a mediation effect found. This exploratory post hoc finding certainly requires replication, as the mediation effect was only found for one of the two criteria (tertiary, rather than secondary academic performance) and one of the two predictors (cognitive ability, rather than curiosity). In addition, the effect size was very small and was found in absence of a direct effect of cognitive ability on performance. Future investigations might nonetheless investigate the benefit of a closer match of behavioral indicators of environmental enrichment and knowledge domains. Generally, matching predictor and criterion variables in terms of bandwidth and fidelity increases the amount of shared variance (Brunswik 1956; Schulze et al. 2021; Wittmann 1988) yet also comes at the expense of knowledge gain when high levels of specificity lead to trivial findings (e.g., learning more for a particular exam leads to a better grade; Funder 2009). Additionally, some of the indicators of epistemic behavior used in the present study might rather reflect habits (e.g., leisure activities) than self-directed knowledge or ability expansion; thus, future research is needed to test the environmental enrichment hypothesis with alternative indicators of epistemic behavior.

The environmental enrichment hypothesis might also have differential meaning according to age. The study by Raine et al. (2002) investigated stimulation-seeking in 3-year-old children in terms of physically exploring their environment to engage socially with other children. Such behavior might have a more pronounced effect in the developing brain of children, compared to a visit to a museum of an adolescent, and effects might yet be different in old age when the influence of an enriched environment rather contributes to an attenuated decline in cognitive functioning (Rouillard et al. 2017; Ziegler et al. 2015).

As a limitation of the present study, the sample size for some of the tested effects, especially for the exploratory analyses implementing a full prospective design, were rather small. As such, it can be speculated that a mediation effect might exist on the population level yet is so small that it can only be detected by studies with a larger sample size. Even then, however, it should be clear that environmental enrichment is certainly not the only mechanism underlying the cognitive consequences of the personality trait curiosity.

The present research was preregistered prior to the collection of data at T3, and the study was conducted and analyzed as closely as possible along these lines. Nonetheless, the data would allow for testing several alternative models and hypotheses that underly the relation of the variables under investigation. For example, according to the mediation model of the OFCI model by Ziegler et al. (2012), the relation between curiosity and knowledge attainment should be mediated by fluid intelligence, indicating that for knowledge attainment it does not suffice that an enriched environment is sought out, but employing fluid abilities to make sense of the stimuli might be a necessary prerequisite. Additionally, the environmental enrichment might also have a moderating (rather than a mediating) influence on the relation between fluid intelligence and academic performance, indicating the relation is stronger for individuals who seek cognitively stimulating environments.

## 5. Conclusions

The present preregistered, longitudinal study investigated in a large and heterogenous sample of young adults the processes underlying the predictive validity of two major antecedents of academic performance, cognitive ability, and curiosity. Based on the environmental enrichment hypotheses, it was predicted that epistemic behaviors mediate this relation. The hypothesis was not confirmed. Alternative approaches, including the investigation of cognitive variables, might shed more light on the processes underlying these relations.

## Figures and Tables

**Table 1:** Bivariate associations between the study variables.

	N	1	2	3	4	5	6	7	8	9
1: Cognitive ability (T1)	1940									
2: Cognitive ability (T2)	1343	0.76 **								
3: Cognitive ability (T3)	420	0.75 **	0.79 **							
4: Curiosity (T1)	1662	0.27 **	0.27 **	0.24 **						
5: Curiosity (T2)	1485	0.15 **	0.19 **	0.18 **	0.57 **					
6: Curiosity (T3)	434	0.15 **	0.25 **	0.17 **	0.53 **	0.69 **				
7: Epistemic behavior (T2)	1190	−0.02	0.00	−0.02	0.27 **	0.38 **	0.24 **			
8: Epistemic behavior (T3)	401	−0.08	0.00	−0.04	0.22 **	0.38 **	0.39 **	0.43 **		
9: Secondary GPA (T3)	421	0.39 **	0.41 **	0.46 **	0.20 **	0.19 **	0.09 *	0.15 **	0.08	
10: Tertiary GPA (T3)	143	0.10	0.08	0.07	0.08	0.10	0.01	0.14	0.05	0.40

Note: GPA: grade point average. *: *p* < 0.05; **: *p* < 0.01.

**Table 2:** Estimated standardized latent regression weights and indirect and total effects.

	a	b	c	Indirect	total
Cognitive ability on secondary GPA	0.05	−0.04	0.51 **	0.00	0.51 **
Cognitive ability on tertiary GPA	0.05	0.03	0.14	0.00	0.14
Curiosity on secondary GPA	0.35 **	0.06	0.23	0.02	0.25 **
Curiosity on tertiary GPA	0.35 **	0.04	0.10	0.01	0.12

Note: For all four models, the mediator was epistemic behavior. **: *p* < 0.01.

## Data Availability

The data, script, and materials are freely available on OSF (https://osf.io/th86q/; accessed on 12 April 2022).

## Appendix A. Deviation from the Preregistration

I preregistered that there were 1089 participants at T2. This is not correct. As noted in the method section, a total of 1679 individuals participated at T2, 1235 of which had valid data on epistemic behavior.

I preregistered the use of five indicators of epistemic behavior, the fifth being complexity of the current education/occupation. This indicator was assessed in 2018 using a single multiple-choice item with the levels (1) working; (2) vocational training; (3) studying at a university of applied sciences; or (4) studying at university. These levels were supposed to reflect increasing levels of complexity and, correspondingly, epistemic demands (“working” was coded as lowest level of complexity as, according to the age of the participants, it was assumed that working right after school without former education is likely to be unskilled work). However, in the context of the present study, this indicator is not a suitable mediator for the prediction of grades. In case of secondary GPA, the indicator cannot be used as predictor because the behavior assessed pertains to occupational choices after having graduated from secondary school. In case of tertiary GPA, the indicator is strongly confounded (r = 0.80) with occupational/university degree (“vocational training” vs. “Bachelor/Master”), which was used to correct for level differences in grades and is, therefore, part of the criterion variable. Thus, this indicator was not used as the fifth indicator of epistemic behavior.

According to the recommendation of an anonymous reviewer, the data were analyzed with a latent change score model with mediation effects, rather than a manifest mediation analysis. However, results for the main hypotheses remain unchanged when the latter analysis was used, as preregistered. Accordingly, the data were analyzed with the R package lavaan (Rosseel 2012) rather than psych (Revelle 2020), which also allows for dealing with missing data via full information maximum likelihood (Enders and Bandalos 2001).

Post hoc power analyses were performed, as preregistered for the mediation model on the manifest level according to a medium effect of 0.39 according to (Zhang and Wang 2013). However, Gignac and Szodorai (2016) provide empirical evidence for typical effect sizes for individual differences which also better correspond to the population effect size in the prediction of academic performance (e.g., Poropat 2009). Thus, power was additionally computed for a = b = 0.20.

I preregistered to exclude participants from T3 with missingness on T2. However, as these participants have valid data on T1, it is better (in terms of statistical power) to include them and to deal with missingness via full information maximum likelihood (Enders and Bandalos 2001). However, results are identical when excluding participants, as preregistered.
